# A comprehensive systematic review and meta-analysis study in comparing decompressive craniectomy versus craniotomy in patients with acute subdural hematoma

**DOI:** 10.1007/s10143-024-02292-5

**Published:** 2024-02-10

**Authors:** Mohammad Amin Habibi, Andrew J. Kobets, Amir Reza Boskabadi, Mehdi Mousavi Nasab, Pooria Sobhanian, Fatemeh Saber Hamishegi, Seyed Ahmad Naseri Alavi

**Affiliations:** 1 Skull Base Research Center, Loghman Hakim Hospital, Shahid Beheshti University of Medical Sciences, Tehran, Iran; 2 Clinical Research Development Center, Qom University of Medical Sciences, Qom, Iran; 3 Department of Neurological Surgery, Montefiore Medical Center, Albert Einstein College of Medicine, Bronx, NY 10467 USA; 4 Faculty of Medicine, Mashhad University of Medical Science, Mashhad, Iran; 5 Faculty of Medicine, Shahid Beheshti University of Medical Sciences, Tehran, Iran; 6 Student Research Committee, Faculty of Medicine, Mazandaran University of Medical Sciences, Sari, Iran; 7 Faculty of Medicine, Guilan University of Medical Science, Rasht, Iran

**Keywords:** Intracranial hemorrhage, SDH, TBI, Craniectomy, Decompressive Craniectomy

## Abstract

There are two controversial surgery methods which are traditionally used: craniotomy and decompressive craniectomy. The aim of this study was to evaluate the efficacy and complications of DC versus craniotomy for surgical management in patients with acute subdural hemorrhage (SDH) following traumatic brain injury (TBI). We conducted a comprehensive search on PubMed, Scopus, Web of Science, and Embase up to July 30, 2023, using the Preferred Reporting Items for Systematic Reviews and Meta-Analyses checklist. Relevant articles were reviewed, with a focus on studies comparing decompressive craniectomy to craniotomy techniques in patients with SDH following TBI. Ten studies in 2401 patients were reviewed. A total of 1170 patients had a craniotomy, and 1231 had decompressive craniectomy. The mortality rate was not significantly different between the two groups (OR: 0.46 [95% CI: 0.42–0.5] *P*-value: 0.07). The rate of revision surgery was insignificantly different between the two groups (OR: 0.59 [95% CI: 0.49–0.69] *P*-value: 0.08). No significant difference was found between craniotomy and decompressive craniectomy regarding unilateral mydriasis (OR: 0.46 [95% CI: 0.35–0.57] *P*-value < 0.001). However, the craniotomy group had significantly lower rates of non-pupil reactivity (OR: 0.27 [95% CI: 0.17–0.41] *P*-value < 0.001) and bilateral mydriasis (OR: 0.59 [95% CI: 0.5–0.66] *P*-value: 0.04). There was also no significant difference in extracranial injury between the two groups, although the odds ratio of significant extracranial injury was lower in the craniotomy group (OR: 0.58 [95% CI: 0.45–0.7] *P*-value: 0.22). Our findings showed that non-pupil and bilateral-pupil reactivity were significantly more present in decompressive craniectomy. However, there was no significant difference between the two groups regarding mortality rate, extracranial injury, revision surgery, and one-pupil reactivity.

## Introduction

Traumatic brain injury (TBI) and stroke are significant public health concerns on a global scale, resulting in severe neurological consequences for patients. Around one-third of individuals with serious TBI exhibit the presence of acute subdural hematomas (ASDHs) [1, 2]. Following TBI and the occurrence of SDH leads to a high mortality rate of 40 to 60%, and a range of 19 to 45% of functional recovery rate [3, 4].

Following TBI and expansion of SDH, a range of neurological deficits would be manifested to increase the intracranial pressure (ICP), and significant mass volume effect [5, 6]. It is also recommended for surgical evacuation in acute SDH if the thickness of the mass is higher than 10 mm or the midline shift is more than 5 mm with any GSC, nonetheless. On the other hand, patients with GCS ≤ 8 are highly recommended for evacuation in any SDH volume [5, 6].

The most often performed surgical procedure for individuals in need of cerebral decompression following a TBI or stroke is a decompressive craniectomy (DC). Craniotomy and DC are often conducted neurological surgeries that are carried out in both elective and emergencies. The term “craniotomy” pertains to the temporary extraction of a bone flap from the calvarium to get access to the intracranial components. DC is a separate procedure that involves the excision of a skull flap without subsequent restoration. In the context of emergency medicine, it is commonly recommended to administer this treatment to alleviate heightened ICPs in individuals with edema of the cerebral cortex, evacuate intracranial hemorrhages, or facilitate the drainage of intracranial abscesses [7].

DC may be performed as a post-surgical procedure following craniotomy to relieve intracranial contents, particularly in cases of skull flap abscesses [8]. Enhancing system compliance through the implementation of an expandable container represents a sensible approach to utilizing DC with no need for bone flap replacement [9]. DC has been employed for a considerable duration in patients with head trauma. However, numerous inquiries remain regarding the efficacy of its implementation in routine clinical settings [10].

Similar to other invasive procedures, DC is associated with well-documented consequences. Some of the observed consequences encompass the occurrence of contusions, herniation of the cerebral tissue outside the cranial cavity, absence of safeguarding against subsequent trauma, and compression of cortical tissue at the outer edge of the bone flap [11].

Numerous studies have documented that the replacement of the bone flap is associated with the reversal of many of these problems that are related to cerebral hemodynamics [12–14] and also syndrome of trephined (SOT) with a poorly known incidence between 1 and 40%. SOT also burdens sensorimotor or cognitive worsening after DC. It will be manifested with dizziness, fatigability, tinnitus, pain discomfort in the site of DC, and psychological issues [15–17].

During the first postoperative phase, radiologists face the challenge of differentiating between multiple entities, as the wide spectrum of usual brain morphologies following craniotomy and DC can make this task difficult. The timely identification of postoperative complications can significantly aid neurosurgeons, hence affording radiologists a pivotal position in the provision of medical services to patients.

The effectiveness, indications, risks, and complications of DC vs. craniectomy in patients with SDH still remain controversial. Hence, the main aim of this study is to assess the effectiveness of DC in comparison to other surgical techniques for cranial conditions in patients with acute SDH. Additionally, this study seeks to evaluate the rate of complications associated with DC and other neurocranial surgeries for TBI and ASDH. Lastly, provide an overview of the postoperative care required for these patients.

### Importance and novelty

There has been a controversial attitude over using craniotomy compared with DC in patients following SDH up to now. As mentioned above, it appears that the ratio of craniotomy is significantly higher than that of DC. However, the clinical outcomes have not demonstrated a better and dramatic difference in some cases. In the present study, we tried to investigate and update the advantages and disadvantages of both techniques to distinguish whether there is any difference in clinical outcomes based on the published papers. The results and conclusion of this study may be helpful for neurosurgeons in selecting the better option based on their advantages and disadvantages and avoiding the overuse of one method.

## Materials and methods

### Object

The systematic review was conducted by the suggested procedures outlined by the Preferred Reporting Items for Systematic Reviews and Meta-Analyses for Protocols (PRISMA) [18].

### Eligibility criteria

The study’s inclusion criteria encompassed randomized controlled trials, prospective and retrospective cohort studies, case–control studies, and cross-sectional studies. Only patients who underwent DC or craniotomy procedures as a result of ASDH following TBI. The research papers must be published in the English language and should explicitly specify the total number of participants who participated in the study. Furthermore, the studies incorporated in the analysis must offer comprehensive and adequate information regarding injuries, surgical procedures, criteria for follow-up, and the utilization of appropriate statistical analysis methods. Research investigations that specifically examine non-subdural hematoma and non-DC surgery will be omitted from consideration.

### Databases and search strategy

To identify pertinent trials, a comprehensive search was conducted across various electronic databases including PubMed, Web of Science, Scopus, and Embase. The search tactics outlined in the protocol (Appendix 1) were employed to collect evidence from original papers published up until July 30, 2023. The searches were performed without any geographical limitation but included only English language publications. The search of databases was conducted using the specific medical subject heading in the search were “Decompressive Craniectomy” OR “Craniectomy” AND “Craniotomy” AND “Acute Subdural Hemorrhage” OR “Subdural Hemorrhage” OR “SDH” OR “Acute Subdural Hematoma” OR “Subdural Hematoma.”

All studies including case reports, letters to editors, review articles, and other relevant data were enrolled in the study. The titles and abstracts of all articles attained from the databases were reviewed by two senior independent authors to investigate eligibility based on the aim of the study. The “Find duplicates” feature of the EndNote software was utilized to eliminate any in All references were imported into Endnote reference software and de-deduplication was performed. They underwent further de-duplication after screening by a team of reviewers and then joint articles.

### Study selection

To ensure the accuracy and relevance of the study, a complete screening process was done by two authors. The initial step was a thorough examination of the titles and abstracts of the selected research, to identify and eliminate any redundant citations or irrelevant sources. Following the aforementioned initial phase, the researchers acquired the comprehensive texts of the remaining citations to facilitate subsequent assessment. Subsequently, both writers conducted individual and meticulous assessments of the complete papers, exclusively choosing those that satisfied the predetermined criteria for inclusion. Excluded from consideration were any procedures, designs, development papers, or opinion pieces. In instances where the two authors held divergent viewpoints, they endeavored to reconcile their disparities through constructive dialogue or sought the counsel of a third author.

To identify more relevant scholarly pieces, a thorough examination was conducted of the references cited within the selected papers. Subsequently, a comprehensive evaluation was conducted on the entirety of the final studies, encompassing an assessment of their quality, data collection, and information analysis.

### Data extraction

Two reviewers conducted the data extraction process. The information on demographic characteristics include author name, study period, country, design of study, total number of patients, gender, follow-up duration, number of patients in the craniotomy and DC groups, gender of patients in the craniotomy and DC groups, and mean age of patients in both groups. The data regarding Glasgow coma score (GCS) 3–8, 9–12, and 13–15, mortality rate, revision surgery rate, significant extracranial injury, and pupil reactivity were also extracted.

### Statistical analysis

The statistical analysis was conducted according to the Cochrane Handbook for systematic reviews of interventions [19]. The odds ratio (OR) was used to compare craniotomy and DC groups with a random-effect model for pooling the outcome. The heterogeneity measured by *I*^2^ statistics and chi-square *P*-value (*χ*^2^), which *I*^2^ statistics > 50% and chi-square *P*-value < 0.05, represents high heterogeneity [20]. All statistical analysis was conducted by STATA V.17. The *P*-value < 0.05 was considered statistically significant.

## Results

### Study selection characteristics

**Fig. 1:**
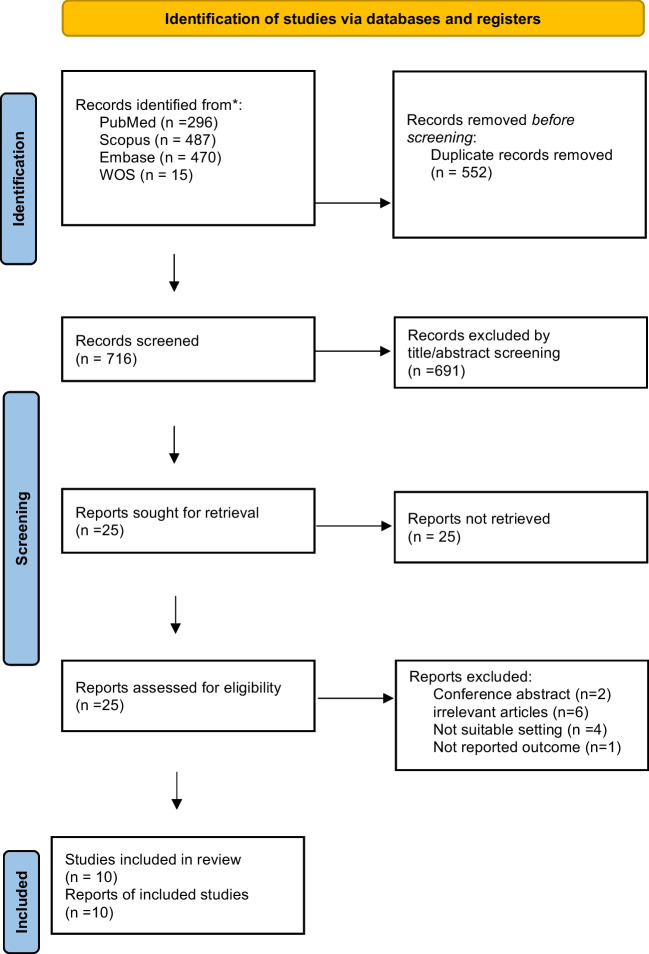
PRISMA flowchart

A total of 1268 articles were identified by a comprehensive search of all recruiting databases, of which 552 duplicate articles were removed from the study selection process. A total of 716 articles were used for title/abstract screening and 25 articles remained for further evaluation. In the full-text assessment, 13 articles were excluded due to the not suitable setting (*n* = 4), irrelevant articles (*n* = 6), not reporting the outcome (*n* = 1), and conference abstract (*n* = 2). Finally, 10 articles [1, 21–29] (Table 1) fully met the eligibility criteria and were used for data synthesis. Figure 1 represents the PRISMA flowchart of the study selection process.

**Table 1:** Demographic characteristics

First author name	Study period	Country	Type of study	No. of patients	Gender	Follow-up (months)	Number of craniotomies	Number of craniectomies	Craniotomy male/female	Craniectomy male/female	Craniotomy age (mean ± SD)	Craniectomy age (mean ± SD)
Woertgen 2006 [1]	1992–2003	Germany	R. cohort	180	113M/67F	61 months	111	69	-	-	57.2	52
Tsermoulas 2016 [2]	2011–2014	UK	R. cohort	99	79M/20F		30	69	22M/8F	57M/12F	48	44
Mohamed Azouz 2023 [3]	2019–2021	Egypt	P. cohort	30	21M/9F	1 month	15	15	-	-	-	-
Rush 2016 [4]	2006–2011	USA	R. cohort	302	214M/88F	-	151	151	106M/45F	108M/43F	52.73	52.37
Li 2012 [5]	2005–2010	UK	R. cohort	91	51M/34F	-	40	51	18M/18F	33M/16F	59	45
Kwon 2016 [6]	2010–2014	South Korea	R. cohort	46	28M/18F	-	20	26	12M/8F	16M/10F	63.4	65.5
Chen 2011 [7]	2000–2009	Taiwan	R. cohort	102	62M/40F	-	42	60	21M/21F	41M/19F	47.4 ± 20.9	41.2 ± 16.4
Hutchinson 2023 [8]	2014–2019	UK	Randomized trial	450	357M/83F	12 months	228	222	178M/40F	179M/43F	48.3 ± 16.5	48.8 ± 16.6
Bemora 2022 [9]	2019–2020	Madagascar	R. cohort	73	63M/10F	-	19	54	-	-	44.16	35.63
Shibahashi 2020 [10]	2004–2015	Japan	R. cohort	1028	-	-	514	514	353M	353M	64	63

### Demographic characteristics

A total of 2401 patients were included which 1170 patients were treated with craniotomy and 1231 patients with DC. The number of male and female genders was reported in seven studies. In the craniotomy group, 710 males and 140 females with a 5.07-fold male-to-female ratio were recruited. In DC, 787 males and 143 females were included with a 5.5-fold male-to-female ratio which highlights a higher proportion of male-to-female in the DC group than craniotomy. The mean age of patients was reported in nine studies, which ranged from 44.16 to 64 years and 35.63 to 65.5 years in the craniotomy and DC groups, respectively. Table 1 represents the demographic characteristics of patients.

**Fig. 2:**
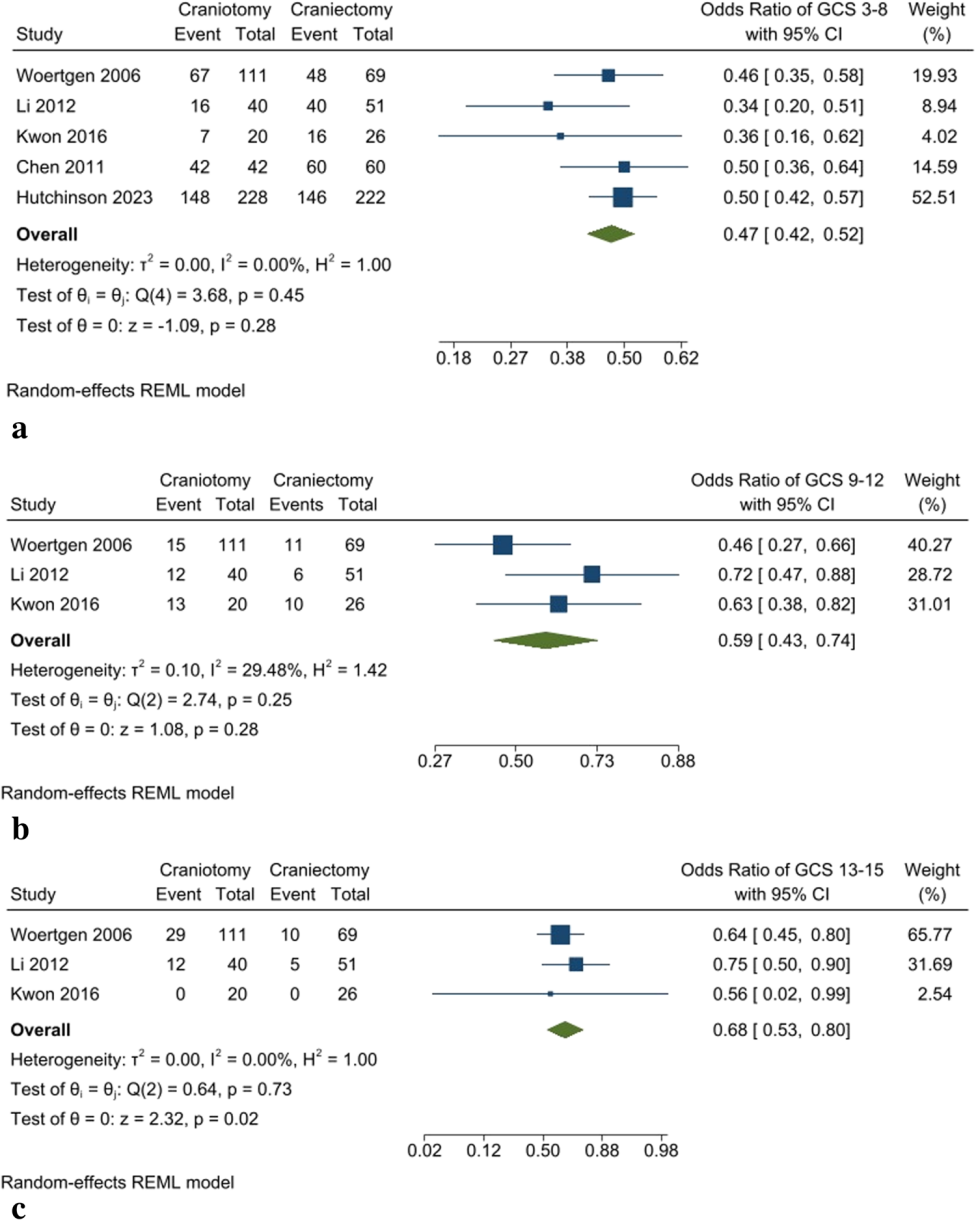
**a** Prevalence of patients with GCS 3–8. **b** Prevalence of patients with GCS 9–12. **c** Prevalence of patients with GCS 13–15

The severity of TBI was measured by the GCS scale. For patients with GCS 3–8, indicating severe TBI, an insignificant lower proportion of GCS 3–8 rate was present in the craniotomy group compared to the craniectomy group (OR: 0.47 [95% CI: 0.42–0.52] *P*-value: 0.28, *I*^2^: 0%, chi-square *P*-value: 0.45) (Fig. 2a). Also, there was a lower rate, but no significant difference in the proportion of GCS 9–12 in the craniotomy group than the DC group (OR: 0.59 [95% CI: 0.43–0.74] *P*-value: 0.25, *I*^2^: 29.48%, chi-square *P*-value: 0.28) (Fig. 2b). However, a significantly lower rate of GCS 13–15 was evident in the craniotomy group than the DC group (OR: 0.68 [95% CI: 0.53–0.8] *P*-value: 0.73, *I*^2^: 0%, chi-square *P*-value: 0.02) (Fig. 2c).

### Mortality

**Fig. 3:**
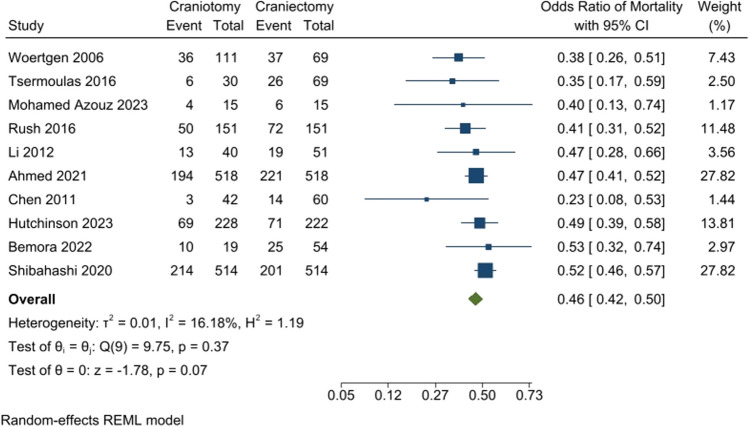
Mortality rate of patients

The mortality rate was compared between the craniotomy and DC groups. It was shown that the mortality rate was significantly lower in the craniotomy group than the DC group (OR: 0.46 [95% CI: 0.42–0.5] *P*-value: 0.07, *I*^2^: 16.18%, chi-square *P*-value: 0.37) (Fig. 3).

### Revision surgery

**Fig. 4:**
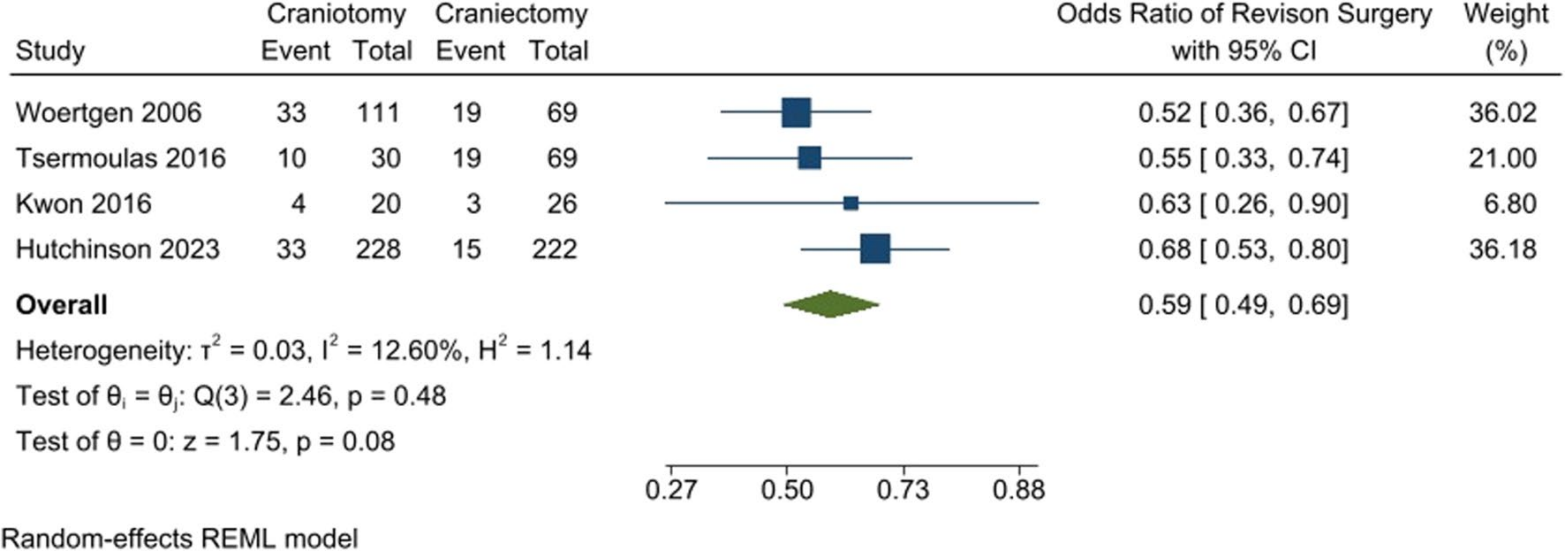
Revision surgery rate

The rate of revision surgery was lower in the craniotomy group than the DC group. The odds of revision surgery were not significantly different between the two groups (OR: 0.59 [95% CI: 0.49–0.69] *P*-value: 0.08, *I*^2^: 12.60%, chi-square *P*-value: 0.48) (Fig. 4).

### Pupil reactivity

**Fig. 5:**
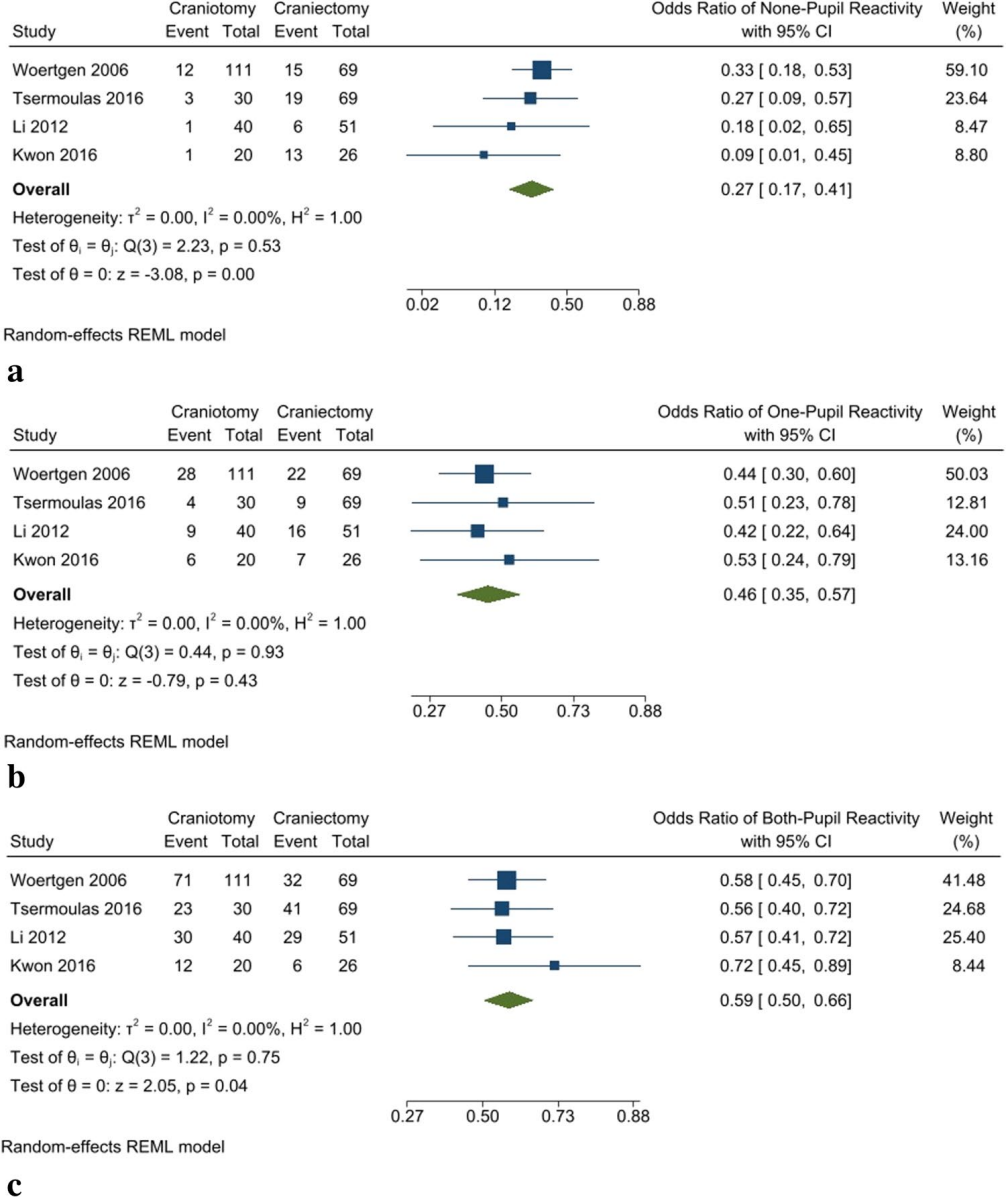
**A** No pupil reactive rate. **b** One-eye pupil reactive rate. **c** Two-eye pupil reactive rate

There was no significant difference between craniotomy and DC regarding unilateral mydriasis (OR: 0.46 [95% CI: 0.35–0.57] *P*-value < 0.001, *I*^2^: 0%, chi-square *P*-value: 0.93) (Fig. 5b). However, there was a significant difference between the two groups and a significantly lower non-pupil reactivity rate (OR: 0.27 [95% CI: 0.17–0.41] *P*-value < 0.001, *I*^2^: 0%, chi-square *P*-value: 0.53) and bilateral mydriasis rate (OR: 0.59 [95% CI: 0.5–0.66] *P*-value: 0.04, *I*^2^: 0%, chi-square *P*-value: 0.75) were evident in the craniotomy group than DC (Fig. 5a and Fig. 5c).

### Significant extracranial injury

**Fig. 6:**
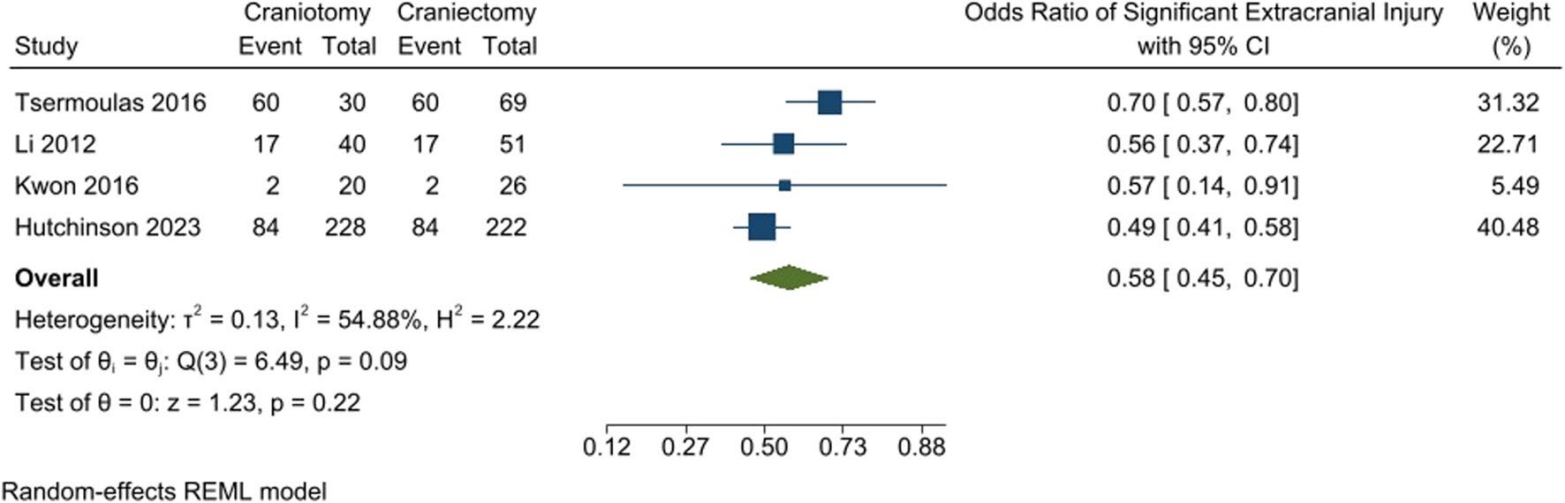
Significant extracranial injury rate

The two groups were compared for significant extracranial injury, which was not significantly different. The odds ratio of significant extracranial injury was lower in the craniotomy group than DC (OR: 0.58 [95% CI: 0.45–0.7] *P*-value: 0.22, *I*^2^: 54.88%, chi-square *P*-value: 0.09) (Fig. 6).

### Publication bias

The publication bias of studies was also evaluated by the regression-egger test. The funnel plots are present in the supplementary file. No significant publication bias was present in GCS 3–8 (*t* =  − 1.50, *P*-value: 0.2295), GCS 9–12 (*t* =  − 1.64, *P*-value: 0.3484), GCS 13–15 (*t* = 0, *P*-value: 0.9983), mortality (*t* =  − 1.90, *P*-value: 0.0942), revision surgery (*t* = 0.04, *P*-value: 0.9711), significant extracranial injury (*t* = 1.82, *P*-value: 0.2101), non-pupil reactivity (*t* =  − 1.39, *P*-value: 0.2988), one-pupil reactivity (*t* = 0.49, *P*-value: 0.6724), and two-pupil reactivity (*t* = 0.92, *P*-value: 0.4529). No significant publication bias suggests that there was no significant impact of publication bias on the outcomes.

## Discussion

Based on our findings, it appears that primary DC is not necessarily associated with better functional outcomes when compared to craniotomy. Patients undergoing DC had higher GCS 13–15 scores than the craniotomy groups. The mortality rate was not significantly different between the two groups. No significant difference was found between craniotomy and DC regarding unilateral mydriasis. However, the craniotomy group had lower rates of non-pupil reactivity and bilateral mydriasis. There was also no significant difference in extracranial injury between the two groups.

A previous meta-analysis study showed that the clinical presentation and postoperative outcome were worse in patients who underwent DC compared with craniotomy. However, DC led to a lower incidence of residual SDH after treatment [30]. Pulmonary complications are well-described in patients with TBI and complications present in patients with TBI and SDH can be even life-threatening. Among 2370 patients with SDH, which included 1852 patients who underwent craniotomy and 518 who underwent DC, there were no significant differences between the two groups regarding acute respiratory distress syndrome, pneumonia, and pulmonary embolism [31]. A multi-center observational study comparing craniotomy and DC showed that DC was associated with more complications than craniotomy, but the in-hospital mortality odds were similar between the two groups [32].

Hutchinson et al. [27] conducted a randomized clinical trial and investigated the disability and quality of life with the EuroQol Group 5-Dimension 5-Level questionnaire (EQ-5D-5L). The EQ-5D-5L scores at 12 months were comparable between the DC and craniotomy groups. Within 2 weeks after randomization, 14.6% of the craniotomy group underwent additional cranial surgery, compared to 6.9% of the DC group. The incidence of wound complications was 3.9% in the craniotomy group and 12.2% in the DC group.

According to previous research, using DC in patients with more severe head injuries is common [24]. Additionally, a meta-analysis suggests a correlation between a lower GCS score and a higher proportion of patients undergoing DC [33]. It is noteworthy that brain swelling is often the reason for including patients in the DC approach [34].

DC offers a theoretical advantage of better control of elevated ICP, improvement in cerebral perfusion pressure, and brain partial pressure of oxygen [35]. Nevertheless, the basis for this procedure is contentious since cerebral edema or raised ICP does not occur in all patients who undergo DC [36]. The management of ICP has an unclear role, as it is not yet established whether elevated ICP is a primary pathological event or a secondary outcome that indicates other underlying processes not affected by ICP management [37]. There have been reports of higher mortality associated with DC compared to craniotomy [38]. This increased mortality could be attributed to axon stretching and delayed intracranial bleeding following persistent bony defects left by the DC procedure [11, 39]. Besides, DC has been linked to cerebral autoregulation and hemodynamic changes [33].

Patients with an acute SDH are often treated with surgical management to minimize the occurrence and impact of secondary brain injury [40]. However, this condition’s morbidity and mortality rates stay high despite surgical intervention. Craniotomy and DC are commonly used surgical approaches for treating acute SDH. However, there is currently no universally accepted consensus on which management strategy is the most appropriate. Ultimately, choosing between these options is left to the surgeon’s discretion. There is a considerable disparity in the approach adopted by various continents, countries, and even individual departments when it comes to this condition. This inconsistency can be attributed to the absence of credible evidence and definitive standards of practice [41].

Primary DC is a recognized treatment for patients with ASDH who have severe brain swelling that prevents the replacement of the bone flap [41, 42]. According to a recent agreement, the bone flap should not be returned if the brain is bulging beyond the inner table of the skull during surgery. DC is advantageous in reducing the elevation of ICP, thus potentially preventing further brain injury and improving clinical outcomes. However, DC requires additional reconstructive surgery, known as cranioplasty, and presents risks associated with bone defects, infections, and bone flap reabsorption [11]. Furthermore, DC is known to change cerebrospinal fluid flow and cerebral blood flow dynamics, both of which improve with the replacement of the bone flap [42].

There have been varying conclusions from studies on the effectiveness of craniotomy and DC for acute SDH. While some studies suggest a higher risk of complications associated with DC, others indicate no significant difference. Several factors have been identified as potential contributors to the outcomes of acute SDH. These factors include age, the time between injury and treatment, abnormalities in the pupils, preoperative GCS score, length of time with heightened ICP, and CT findings such as midline shift, basal cistern compression, hematoma size, and systemic abnormalities like hypoxia and hypotension [43–45].

## Limitations

Multiple limitations constrain the present study. Baseline characteristics and injury severity, identified as significant confounders in the present analysis, were not adjusted for in the included studies, which is a major limitation. We tried to select the papers only enrolled in patients with GCS ≤ 8 or volume size less than 10 mm. Additionally, the available data did not include information on bone flap size, which can affect surgery outcomes for acute SDH. A randomized study has reported that large bone flaps (> 12 cm) control brain swelling better but are associated with higher complications compared to more minor bone flaps (< 8 cm). Furthermore, it is worth noting that most studies did not provide details of important factors. These factors may differ between institutions and studies, making it challenging to draw definitive conclusions. Additionally, the retrospective nature of most studies included in the analysis may be prone to selection bias and unadjusted for potential confounding variables. It is also essential to consider the impact of including small series or single-center studies on the generalizability of the results and the interpretation of national trends.

## Conclusion

Based on the findings of the study, it is shown that there was no significant difference in mortality rate, revision surgery, one-pupil reactivity, and extracranial injury between the craniotomy and DC groups in patients with SDH. However, the odds ratios for non-pupil and bilateral-pupil reactivity were significantly higher in DC. In other words, non-pupil and bilateral-pupil reactivity were significantly more present in DC.

## Data Availability

None.

## References

[1] Woertgen C, Rothoerl RD, Schebesch KM, Albert R (2006) Comparison of craniotomy and craniectomy in patients with acute subdural haematoma. J Clin Neurosci 13(7):718–721 16904897 10.1016/j.jocn.2005.08.019

[2] van Essen TA, Lingsma HF, Pisică D, Singh RD, Volovici V, den Boogert HF et al (2022) Surgery versus conservative treatment for traumatic acute subdural haematoma: a prospective, multicentre, observational, comparative effectiveness study. Lancet Neurol 21(7):620–631 35526554 10.1016/S1474-4422(22)00166-1

[3] Hanif S, Abodunde O, Ali Z, Pidgeon C (2009) Age related outcome in acute subdural haematoma following traumatic head injury. Irish Med J 102:255–257 19873866

[4] Tallon JM, Ackroyd-Stolarz S, Karim SA, Clarke DB (2008) The epidemiology of surgically treated acute subdural and epidural hematomas in patients with head injuries: a population-based study. Can J Surg 51:339–345 18841222 PMC2556533

[5] Carney N, Totten AM, Reilly C, Ullman JS, Hawryluk GW, Bell MJ, Bratton SL, Chesnut R, Harris OA, Kissoon N, Rubiano AM, Shutter L, Tasker RC, Vavilala MS, Wilberger J, Wright DW, Ghajar J (2017) Guidelines for the management of severe traumatic brain injury, fourth edition. Neurosurgery 80(1):6–15. 10.1227/NEU.0000000000001432 27654000 10.1227/NEU.0000000000001432

[6] Bullock MR, Chesnut R, Ghajar J, Gordon D, Hartl R, Newell DW, Servadei F, Walters BC, Wilberger JE (2006) Surgical management of traumatic brain injury author group Surgical management of acute epidural hematomas. Neurosurgery 58(3 Suppl):S7-15 16710967

[7] Donovan DJ, Moquin RR, Ecklund JM (2006) Cranial burr holes and emergency craniotomy: review of indications and technique. Mil Med 171(1):12–19 16532867 10.7205/milmed.171.1.12

[8] Gooch MR, Gin GE, Kenning TJ, German JW (2009) Complications of cranioplasty following decompressive craniectomy: analysis of 62 cases. Neurosurg Focus 26(6):E9 19485722 10.3171/2009.3.FOCUS0962

[9] Hase U, Reulen H-J, Meinig G, Schürmann K (1978) The influence of the decompressive operation on the intracranial pressure and the pressure-volume relation in patients with severe head injuries. Acta Neurochir 45:1–13 742427 10.1007/BF01774379

[10] Kolias AG, Adams H, Timofeev I, Czosnyka M, Corteen EA, Pickard JD et al (2016) Decompressive craniectomy following traumatic brain injury: developing the evidence base. Br J Neurosurg 30(2):246–250 26972805 10.3109/02688697.2016.1159655 PMC4841020

[11] Stiver SI (2009) Complications of decompressive craniectomy for traumatic brain injury. Neurosurg Focus 26(6):E7 19485720 10.3171/2009.4.FOCUS0965

[12] Erdogan E, Düz B, Kocaoglu M, Izci Y, Sirin S, Timurkaynak E (2003) The effect of cranioplasty on cerebral hemodynamics: evaluation with transcranial Doppler sonography. Neurol India 51(4):479 14742926

[13] Kuo J-R, Wang C-C, Chio C-C, Cheng T-J (2004) Neurological improvement after cranioplasty—analysis by transcranial Doppler ultrasonography. J Clin Neurosci 11(5):486–489 15177389 10.1016/j.jocn.2003.06.005

[14] Richaud J, Boetto S, Guell A, Lazorthes Y (1985) Effects of cranioplasty on neurological function and cerebral blood flow. Neurochirurgie 31(3):183–188 4033856

[15] Honeybul S (2010) Complications of decompressive craniectomy for head injury. J Clin Neurosci 17:430–435. 10.1016/j.jocn.2009.09.007 20181482 10.1016/j.jocn.2009.09.007

[16] Rienzo AD, Colasanti R, Gladi M, Pompucci A, Costanza MD, Paracino R, Esposito D, Iacoangeli M (2020) Sinking flap syndrome revisited: the who, when and why. Neurosurg Rev 43:323–335. 10.1007/s10143-019-01148-7 31372915 10.1007/s10143-019-01148-7

[17] Ashayeri K, Jackson EM, Huang J, Brem H, Gordon CR (2016) Syndrome of the trephined: a systematic review. Neurosurgery 79:525–534. 10.1227/neu.0000000000001366 27489166 10.1227/NEU.0000000000001366

[18] Liberati A, Altman DG, Tetzlaff J, Mulrow C, Gøtzsche PC, Ioannidis JP et al (2009) The PRISMA statement for reporting systematic reviews and meta-analyses of studies that evaluate health care interventions: explanation and elaboration. Annals Int Med 151(4):W-65-W−94 10.7326/0003-4819-151-4-200908180-00136 19622512

[19] Higgins JPT, Thomas J, Chandler J, Cumpston M, Li T, Page MJ, Welch VA (eds) (2023) Cochrane Handbook for Systematic Reviews of Interventions version 6.4 (updated August 2023). Cochrane. Available from http://www.training.cochrane.org/handbook 10.1002/14651858.ED000142 PMC10284251 31643080

[20] Higgins JP, Thompson SG, Deeks JJ, Altman DG (2003) Measuring inconsistency in meta-analyses. BMJ 327(7414):557–560 12958120 10.1136/bmj.327.7414.557 PMC192859

[21] Tsermoulas G, Shah O, Wijesinghe HE, Silva AHD, Ramalingam SK, Belli A (2016) Surgery for acute subdural hematoma: replace or remove the bone flap? World Neurosurg 88:569–575 26523763 10.1016/j.wneu.2015.10.045

[22] Azouz HM, Soffar HMH, Abbass WA et al (2023) Comparative study between the outcome of decompressive craniotomy versus craniectomy in the management of acute subdural hematoma. Egypt J Neurosurg 38:30. 10.1186/s41984-023-00209-w

[23] Rush B, Rousseau J, Sekhon MS, Griesdale DE (2016) Craniotomy versus craniectomy for acute traumatic subdural hematoma in the United States: a national retrospective cohort analysis. World Neurosurg 88:25–31 26748175 10.1016/j.wneu.2015.12.034 PMC4833577

[24] Li LM, Kolias AG, Guilfoyle MR, Timofeev I, Corteen EA, Pickard JD et al (2012) Outcome following evacuation of acute subdural haematomas: a comparison of craniotomy with decompressive craniectomy. Acta Neurochir (Wien) 154(9):1555–1561 22752713 10.1007/s00701-012-1428-8

[25] Kwon YS, Yang KH, Lee YH (2016) Craniotomy or decompressive craniectomy for acute subdural hematomas: surgical selection and clinical outcome. Korean J Neurotrauma 12(1):22–27 27182498 10.13004/kjnt.2016.12.1.22 PMC4866560

[26] Chen SH, Chen Y, Fang WK, Huang DW, Huang KC, Tseng SH (2011) Comparison of craniotomy and decompressive craniectomy in severely head-injured patients with acute subdural hematoma. J Trauma-Injury Infect Crit Care 71(6):1632–1636 10.1097/TA.0b013e3182367b3c 22027888

[27] Hutchinson PJ, Adams H, Mohan M, Devi BI, Uff C, Hasan S et al (2023) Decompressive craniectomy versus craniotomy for acute subdural hematoma. N Engl J Med 388(24):2219–2229 37092792 10.1056/NEJMoa2214172

[28] Ruggeri AG, Cappelletti M, Tempestilli M, Fazzolari B, Delfini R (2022) Surgical management of acute subdural hematoma: a comparison between decompressive craniectomy and craniotomy on patients treated from 2010 to the present in a single center. J Neurosurg Sci 66(1):22–27 30259718 10.23736/S0390-5616.18.04502-2

[29] Shibahashi K, Sugiyama K, Tomio J, Hoda H, Morita A (2020) In-hospital mortality and length of hospital stay with craniotomy versus craniectomy for acute subdural hemorrhage: a multicenter, propensity score-matched analysis. J Neurosurg 133(2):504–513 31226690 10.3171/2019.4.JNS182660

[30] Phan K, Moore JM, Griessenauer C, Dmytriw AA, Scherman DB, Sheik-Ali S et al (2017) Craniotomy versus decompressive craniectomy for acute subdural hematoma: systematic review and meta-analysis. World Neurosurg 101:677–85.e2 28315797 10.1016/j.wneu.2017.03.024

[31] Ahmed N, Greenberg P, Shin SH (2020) Pulmonary complications and sepsis following severe acute subdural hematoma in patients who underwent craniotomy versus craniectomy: a propensity score matched analysis. J Neurol Surg Part A-Central Eur Neurosurg 81(04):297–301 10.1055/s-0040-1701235 32126574

[32] van Essen TA, van Erp IAM, Lingsma HF, Pisică D, Yue JK, Singh RD et al (2023) Comparative effectiveness of decompressive craniectomy versus craniotomy for traumatic acute subdural hematoma (CENTER-TBI): an observational cohort study. EClinMed 63:102161 10.1016/j.eclinm.2023.102161 PMC10432786 37600483

[33] Bor-Seng-Shu E, Figueiredo EG, Amorim RL, Teixeira MJ, Valbuza JS, de Oliveira MM et al (2012) Decompressive craniectomy: a meta-analysis of influences on intracranial pressure and cerebral perfusion pressure in the treatment of traumatic brain injury. J Neurosurg 117(3):589–596 22794321 10.3171/2012.6.JNS101400

[34] Nguyen HS, Janich K, Sharma A, Patel M, Mueller W (2016) To retain or remove the bone flap during evacuation of acute subdural hematoma: factors associated with perioperative brain edema. World Neurosurg 95:85–90 27476687 10.1016/j.wneu.2016.07.067

[35] Howard JL, Cipolle MD, Anderson M, Sabella V, Shollenberger D, Li PM et al (2008) Outcome after decompressive craniectomy for the treatment of severe traumatic brain injury. J Trauma 65(2):380–5 18695475 10.1097/TA.0b013e31817c50d4

[36] Bor-Seng-Shu E, Figueiredo EG, Fonoff ET, Fujimoto Y, Panerai RB, Teixeira MJ (2013) Decompressive craniectomy and head injury: brain morphometry, ICP, cerebral hemodynamics, cerebral microvascular reactivity, and neurochemistry. Neurosurg Rev 36(3):361–370 23385739 10.1007/s10143-013-0453-2

[37] Chesnut RM, Temkin N, Carney N, Dikmen S, Rondina C, Videtta W et al (2012) A trial of intracranial-pressure monitoring in traumatic brain injury. N Engl J Med 367(26):2471–2481 23234472 10.1056/NEJMoa1207363 PMC3565432

[38] Vilcinis R, Bunevicius A, Tamasauskas A (2017) The association of surgical method with outcomes of acute subdural hematoma patients: experience with 643 consecutive patients. World Neurosurg 101:335–342 28216211 10.1016/j.wneu.2017.02.010

[39] Kinoshita T, Yoshiya K, Fujimoto Y, Kajikawa R, Kiguchi T, Hara M et al (2016) Decompressive craniectomy in conjunction with evacuation of intracranial hemorrhagic lesions is associated with worse outcomes in elderly patients with traumatic brain injury: a propensity score analysis. World Neurosurg 89:187–192 26851740 10.1016/j.wneu.2016.01.071

[40] Sawauchi S, Murakami S, Ogawa T, Abe T (2007) Mechanism of injury in acute subdural hematoma and diffuse brain injury: analysis of 587 cases in the Japan Neurotrauma Data Bank. No Shinkei Geka 35(7):665–671 17633509

[41] Hutchinson PJ, Kolias AG, Tajsic T, Adeleye A, Aklilu AT, Apriawan T et al (2019) Consensus statement from the International Consensus Meeting on the Role of Decompressive Craniectomy in the management of traumatic brain injury: consensus statement. Acta Neurochir 161(7):1261–1274 31134383 10.1007/s00701-019-03936-y PMC6581926

[42] Halani SH, Chu JK, Malcolm JG, Rindler RS, Allen JW, Grossberg JA et al (2017) Effects of cranioplasty on cerebral blood flow following decompressive craniectomy: a systematic review of the literature. Neurosurgery 81(2):204–216 28368505 10.1093/neuros/nyx054

[43] d’Avella D, Servadei F, Scerrati M, Tomei G, Brambilla G, Massaro F et al (2003) Traumatic acute subdural haematomas of the posterior fossa: clinicoradiological analysis of 24 patients. Acta Neurochir 145(12):1037–44 14663560 10.1007/s00701-003-0150-y

[44] Hlatky R, Valadka AB, Goodman JC, Robertson CS (2007) Evolution of brain tissue injury after evacuation of acute traumatic subdural hematomas. Neurosurgery 61(1 Suppl):249–54 18813164 10.1227/01.neu.0000279220.30633.45

[45] van Leeuwen N, Lingsma HF, Perel P, Lecky F, Roozenbeek B, Lu J et al (2012) Prognostic value of major extracranial injury in traumatic brain injury: an individual patient data meta-analysis in 39,274 patients. Neurosurgery 70(4):811–8 21904253 10.1227/NEU.0b013e318235d640

